# Interferon β protects against lethal endotoxic and septic shock through SIRT1 upregulation

**DOI:** 10.1038/srep04220

**Published:** 2014-02-27

**Authors:** Chae-Hwa Yoo, Ji-Hyun Yeom, Jin-Ju Heo, Eun-Kyung Song, Sang-Il Lee, Myung-Kwan Han

**Affiliations:** 1 Department of Microbiology, Chonbuk National University Medical School, Jeonju 561-182, Republic of Korea; 2 Institute for Medical Science, Chonbuk National University Medical School, Jeonju 561-182, Republic of Korea; 3 Department of Internal Medicine, Institute of Health Science, Gyeongsang National University College of Medicine, Jinju 660-702, Republic of Korea; 4 These authors contributed equally to this work.

## Abstract

Lipopolysaccharide (LPS), an endotoxin derived from gram-negative bacteria, promotes the secretion of proinflammatory cytokines and mediates endotoxemia through activation of mitogen activated protein kinases, NF-κB, and interferon regulatory factor-3. Silent information regulator transcript-1 (SIRT1), an NAD-dependent deacetylase, mediates NF-κB deacetylation, and inhibits its function. SIRT1 may affect LPS-mediated signaling pathways and endotoxemia. Here we demonstrate that SIRT1 blocks LPS-induced secretion of interleukin 6 and tumor necrosis factor α in murine macrophages, and protects against lethal endotoxic and septic shock in mice. We also demonstrate that interferon β increases SIRT1 expression by activating the Janus kinase – signal transducer and activator of transcription (JAK-STAT) pathway in mouse bone marrow derived macrophages. *In vivo* treatment of interferon β protects against lethal endotoxic and septic shock, which is abrogated by infection with dominant negative SIRT1-expressing adenovirus. Our work suggests that both SIRT1 and SIRT1-inducing cytokines are useful targets for treating patients with sepsis.

Sepsis is defined as a systemic inflammatory response syndrome mediated by a harmful host immune response to infection. Lipopolysaccharide (LPS), a component of the outer membrane of gram-negative bacteria, is a common cause of sepsis via the activation of various immune cells, including monocytes and macrophages^1^,^2^.

Triggering toll-like receptor 4 (TLR4) signaling pathways by LPS as a critical step in the pathogenesis of sepsis leads to the activation of NF-κB and subsequent regulation of immune and inflammatory genes^3^. All TLRs and IL-1 receptor family members have a conserved stretch of approximately 200 amino acids known as the Toll/Interleukin-1 receptor (TIR) domain^4^. Upon stimulation, TLRs associate with many cytoplasmic adaptor proteins containing TIR domains, including myeloid differentiation factor 88 (MyD88), MyD88-adaptor-like/TIR-associated protein (MAL/TIRAP), TIR-domain-containing adaptor-inducing interferon(IFN)-β (TRIF), and Toll-receptor-associated molecule (TRAM)^5^,^6^. This association results in the recruitment and activation of IRAK1 and IRAK4, which form a complex with TRAF6 to activate TAK1 and IKK^5^. IKK activation induces the degradation of IκB and activation of NF-κB.

TLR4 signaling uses at least two distinct pathways and unique downstream adaptor proteins containing TIR domains. TRIF and TRIF-related adaptor molecule mediate MyD88-independent (or TRIF-dependent) signaling pathway, whereas MyD88 and TIR domain-containing adaptor protein mediate MyD88-dependent signaling pathway^7^,^8^. The MyD88-dependent pathway, which is common to all TLRs except TLR3, leads to the activation of interferon regulatory factor (IRF)-5, mitogen activated protein kinase (MAPK), NF-κB, and AP-1. This results in the expression of proinflammatory chemokines and cytokines such as tumor necrosis factor (TNF-α), interleukin-1 (IL-1), IL-6, and IL-β. The MyD88-independent pathway mediates IRF-3 and induces type I IFNs such as IFN-β, CCL5 and CXCL10^9^,^10^,^11^.

Silent information regulator transcript-1 (SIRT1) is an NAD^+^-dependent nuclear histone deacetylase that regulates a variety of physiological processes such as lifespan extension by calorie restriction, cell cycle, metabolism, senescence, apoptosis, inflammation, epigenetic gene silencing by deacetylating histones and transcriptional modulation of gene expression by deacetylaing non-histone proteins,including transcription factors^12^,^13^,^14^,^15^. The non-histone proteins deacetylated by SIRT1 are p53^12^,^16^, NF-κB^15^, peroxisome proliferator-activated receptor γ coactivator 1 α^17^, and forkhead box O3^18^. SIRT1 deacetylates Lys310 of RelA/p65 subunits of NF-κB and inhibits both its transcriptional activity and the release of inflammatory cytokines mediated by NF-κB^15^, suggesting that SIRT1 may regulate the macrophage inflammatory response.

SIRT1 inhibits NF-κB transactivational activity by deacetylation^15^, and inhibits cigarette smoke-induced release of proinflammatory mediator via NF-κB inhibition in response to TNF-α^19^. In addition, SIRT1 knockdown in mouse macrophages activates c-Jun N-terminal kinases (JNK) and IκB kinase inflammatory pathways, and increases TNF-α secretion and SIRT1 activator, leading to the inhibition of other LPS-stimulated inflammatory pathways, as well as secretion of TNF-α^20^,^21^. These suggest that SIRT1 is involved in inflammatory response regulation. In this study, we have demonstrated that LPS induces SIRT1 expression via the activation of the Janus kinase – signal transducer and activator of transcription (JAK-STAT) signaling pathway. Activation of the JAK-STAT signaling pathway is mediated by IFN-β, which is secreted in response to LPS. In addition, we showed that SIRT1 overexpression and IFN-β protect against lethal septic shock by SIRT1 upregulation.

## Results

### LPS induces SIRT1 expression in BMDMs through JAK-STAT signaling pathway

**Figure 1:**
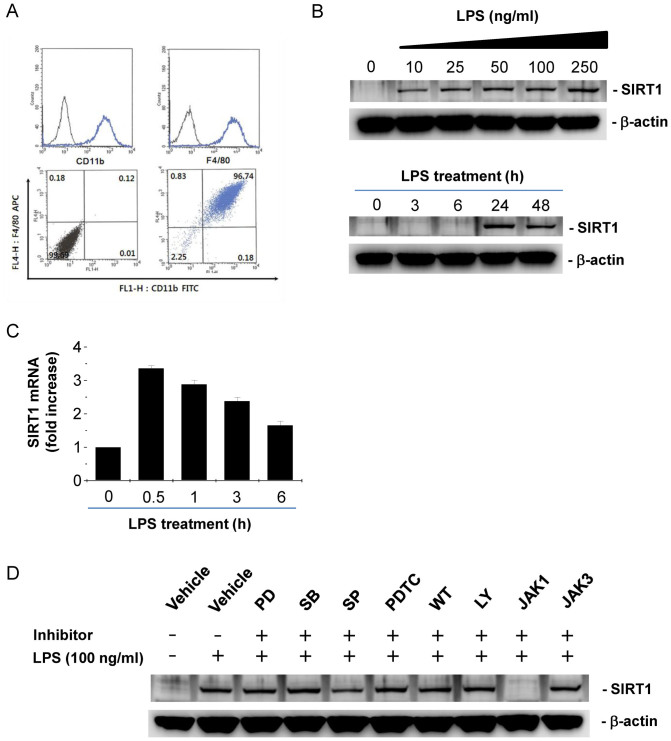
LPS induces SIRT1 expression through JAK-STAT signaling pathway in BMDMs. (A) Phenotypic characterization of BMDMs by flow cytometry. Mouse BMDMs were analyzed by flow cytometry for CD11b and F4/80 cell surface markers to identify the differentiation of bone marrow cells into macrophages. Over 95% of cells within cultures are double positive. (B) Dose- and time- dependant effect of LPS on SIRT1 protein expression. After BMDMs were incubated with various doses of LPS for 24 h (upper panel) and 100 ng/ml LPS for the indicated times (lower panel), western blot were performed with β-actin as a loading control. (C) Time-dependant effect of LPS on SIRT1 mRNA expression. After BMDMs were incubated with 100 ng/ml LPS for the indicated times, real-time PCR were performed. (D) The effects of signal transduction inhibitors on LPS-induced SIRT1 expression. BMDMs were pretreated with 10 μM PD098059 (PD), 10 μM SB203580 (SB), 10 μM SP600125 (SP), 10 μM PDTC, 1 μM wortmannin (WT), 5 μM LY294002 (LY), 5 μM JAK1 inhibitor (JAK1), and 10 μM JAK3 inhibitor (JAK3), for 1 h and further treated with LPS for 24 h. Full-length blots are presented in Supplementary Figure S1–S2. The Western blot shown is a representative of three independent experiments.

We tested whether BMDMs express SIRT1 and, if so, whether LPS changes SIRT1 expression. We differentiated bone marrow cells into macrophages by treatment with M-CSF for 7 days. Over 95% of the BMDMs were double positive for CD11b and F4/80, macrophage surface markers (Fig. 1A), indicating successful differentiation into macrophages. LPS increased SIRT1 expression in a dose dependent manner (Fig. 1B, upper) and LPS-induced SIRT1 expression was maximum at 24 h after the treatment (Fig. 1B, lower). Real-time PCR analysis showed that there was a 4-fold induction of SIRT1 mRNA expression at 6 h after LPS exposure, but the RNA level gradually decreased after that (Fig. 1C). To elucidate the mechanism of SIRT1 expression induced by LPS stimulation, we examined the effects of several inhibitors of signal transduction involved in LPS stimulation. Pretreatment with PD098059, an ERK inhibitor, SB203580, a p38 inhibitor, SP600125, a JNK inhibitor, PDTC, an NF-κB inhibitor, and, wortmannin and LY294002, PI3K inhibitors, did not alter LPS-induced SIRT1 expression (Fig. 1D). However, JAK1 inhibitor abrogated LPS-induced SIRT1 expression (Fig. 1D). These results suggest that LPS-induced SIRT1 expression results from the activation of the JAK-STAT pathway.

### IFN-β signaling is required for LPS-stimulated SIRT1 expression

**Figure 2:**
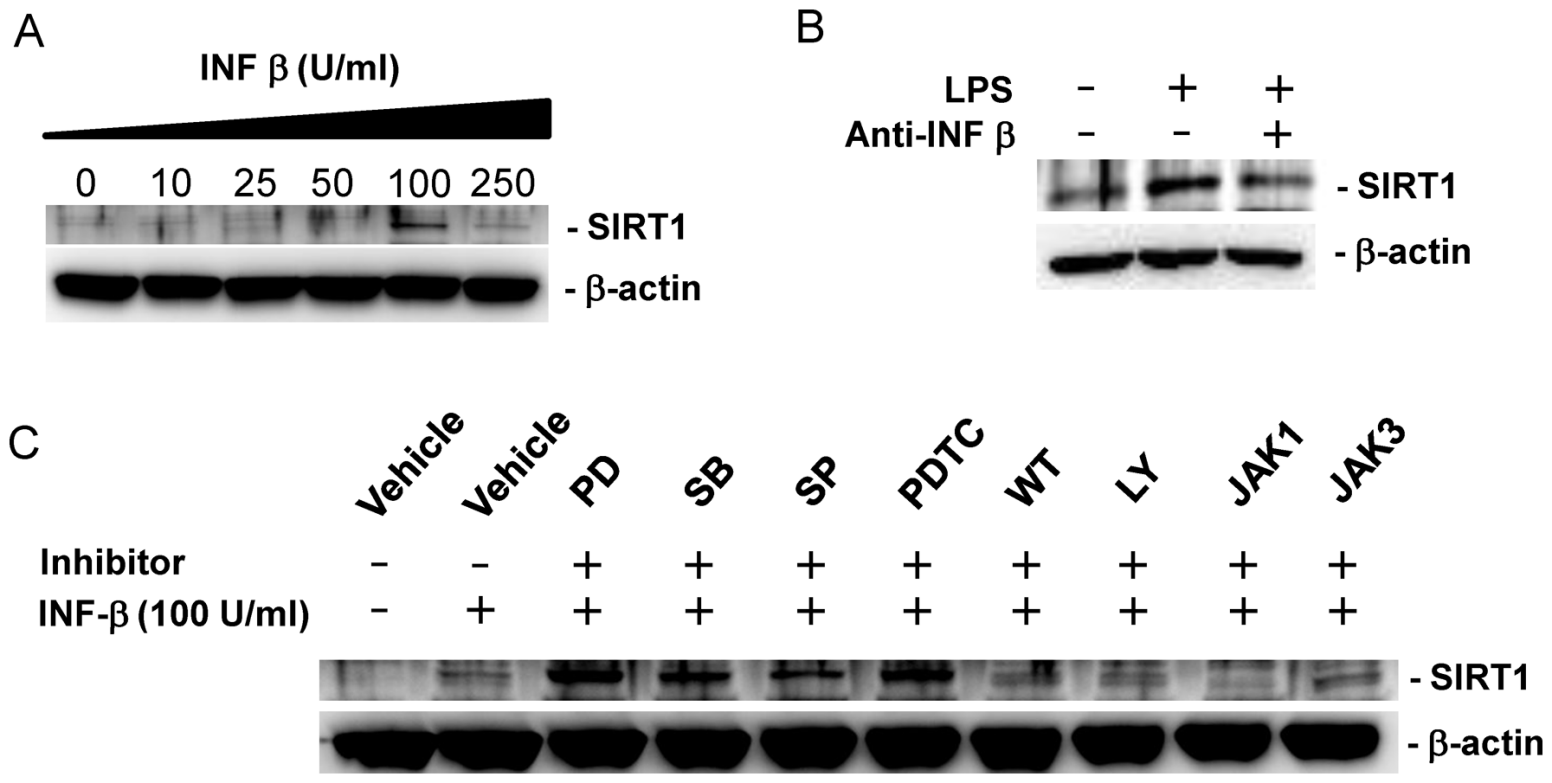
LPS increases SIRT1 expression by IFN-β mediated signaling pathway in BMDMs. (A) Dose dependant effect of IFN-β on SIRT1 protein expression. BMDMs were incubated with the indicated doses of IFN-β for 24 h. A western blot was performed for SIRT1 with β-actin as a loading control. (B) The effect of IFN-β neutralizing antibody on LPS-induced SIRT1 expression. BMDMs were treated with LPS in the presence or absence of IFN-β neutralizing antibody (1 μg/mL) for 24 h. (C) The effect of various inhibitors for signal transduction on IFN-β-induced SIRT1 expression. BMDMs were pretreated with vehicle (DMSO), 10 μM PD098059 (PD), 10 μM SB203580 (SB), 10 μM SP600125 (SP), 10 μM PDTC, 1 μM wortmannin (WT), 5 μM LY294002 (LY), 5 μM JAK1 inhibitor (JAK1), and 10 μM JAK3 inhibitor (JAK3) for 1 h and further treated with 100 U/ml IFN-β for 24 h. Full-length blots are presented in Supplementary Figure S3–S5. The Western blot shown is a representative of three independent experiments.

It has been demonstrated that LPS induces IFN-β expression via the activation of the TLR4 signaling pathway, leading to the activation of the JAK-STAT signaling pathway^22^. This suggests the possibility that LPS induces SIRT1 by IFN-β mediated activation of JAK-STAT signaling pathway. IFN-β treatment induced SIRT1 expression at the dose of 100 U/ml (Fig. 2A). IFN-β-neutralizing antibody blocked LPS-induced SIRT1 expression (Fig. 2B), demonstrating that IFN-β mediates LPS-induced SIRT1 expression. Pretreatment with PD098059, SB203580, SP600125 and PDTC increased IFN-β-induced SIRT1 expression, suggesting negative regulation of IFN-β-induced SIRT1 expression by MAPKs and NF-kB in BMDMs (Fig. 2C). Pretreatment with wortmannin, LY294002 or JAK3 inhibitor did not alter IFN-β-induced SIRT1 expression (Fig. 2C). However, pretreatment with JAK1 inhibitor abrogated IFN-β-induced SIRT1 expression (Fig. 2C). These results suggest that, like LPS-induced SIRT1 expression, IFN-β is a downstream signal that leads to SIRT1 expression through the activation of the JAK-STAT.

### The ectopic expression of SIRT1 or treatment with IFN-β inhibits LPS-induced release of the proinflammatory cytokines TNF-α and IL-6 in BMDMs

**Figure 3:**
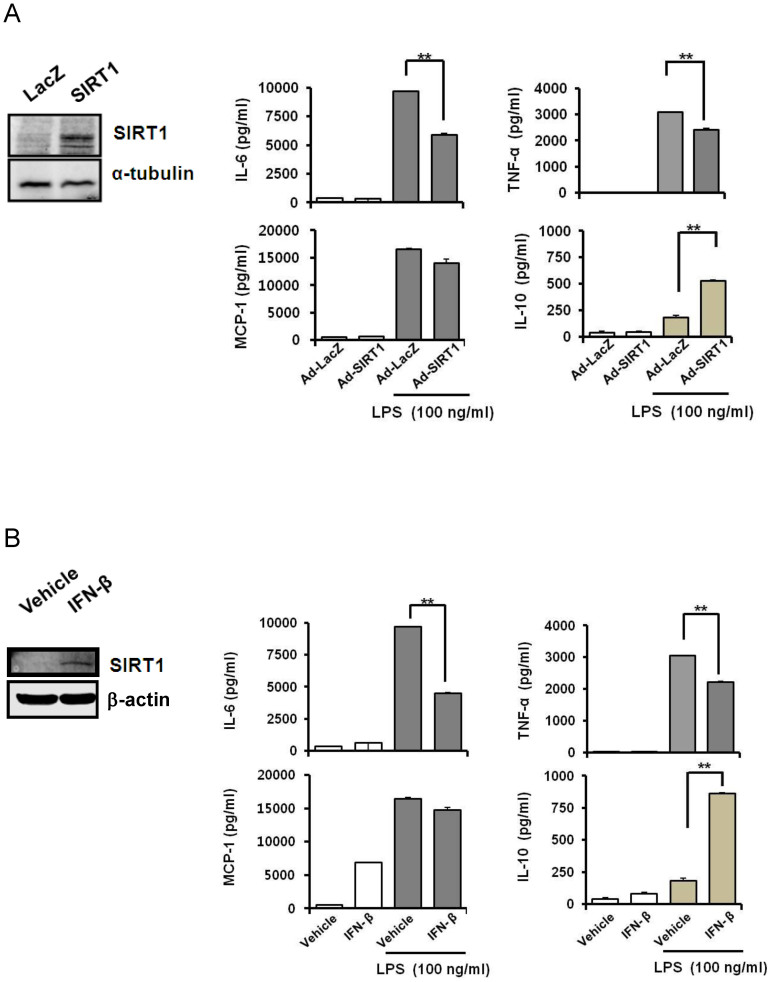
SIRT1 and IFN-β inhibit LPS-induced release of inflammatory cytokines. (A) The effect of SIRT1 expression by adenovirus-SIRT1 on the LPS-induced release of inflammatory cytokines. BMDMs were infected with adenovirus-SIRT1 (10,000 MOI) for 24 h, and then incubated with new media supplemented with LPS (100 ng/mL) for 24 h. Western blotting was performed for SIRT1 using α-tubulin as a loading control (left panel). The levels of several inflammatory cytokines in the cell culture supernatants were measured by ELISA (right panel). (B) The effect of IFN-β treatment on LPS-induced inflammatory cytokine release. BMDMs were pretreated with 100 U/mL IFN-β for 24 h, and then supplemented with LPS (100 ng/mL) for 24 h. Western blotting was performed for SIRT1 using α-tubulin as a loading control (left panel). The levels of several inflammatory cytokines in cell culture supernatants were measured by ELISA (right panel). The results are represented as means ± SEM, * *P* < 0.05, and ** *P* < 0.01. Full-length blots are presented in Supplementary Figure S6–S7. The Western blot shown is a representative of three independent experiments.

SIRT1 is involved in anti-inflammatory responses^20^,^21^. These and our results suggest that SIRT1 may regulate the excessive immune reaction elicited by LPS. To examine whether SIRT1 inhibits LPS-induced release of proinflammatory cytokines, we ectopically expressed SIRT1 in BMDMs using SIRT1 expressing adenovirus (Fig. 3A, left). LPS induces a significantly higher release of TNF-α, IL-6, and MCP-1 (Fig. 3A, right). The ectopic expression of SIRT1 in BMDMs significantly inhibited LPS-induced release of TNF-α and IL-6, but not MCP-1 (Fig. 3A, right). IFN-β also significantly inhibited LPS-induced release of TNF-α and IL-6, but not of MCP-1 (Fig. 3B). We also examined the effect of SIRT1 expression and IFN-β treatment on the LPS-induced release of IL-10 in BMDMs, because IL-10 is a typical anti-inflammatory cytokine that is induced by various stimuli. The elevated level of IL-10 induced by LPS stimulation was additionally increased by SIRT1 overexpression or by IFN-β treatment (Fig. 3A and B). These data indicate that SIRT1 and IFN-β contribute to negative modulation of LPS-mediated inflammatory signaling in BMDMs.

### *In vivo* expression of SIRT1 and treatment with IFN-β improves the survival of LPS-challenged mice

**Figure 4:**
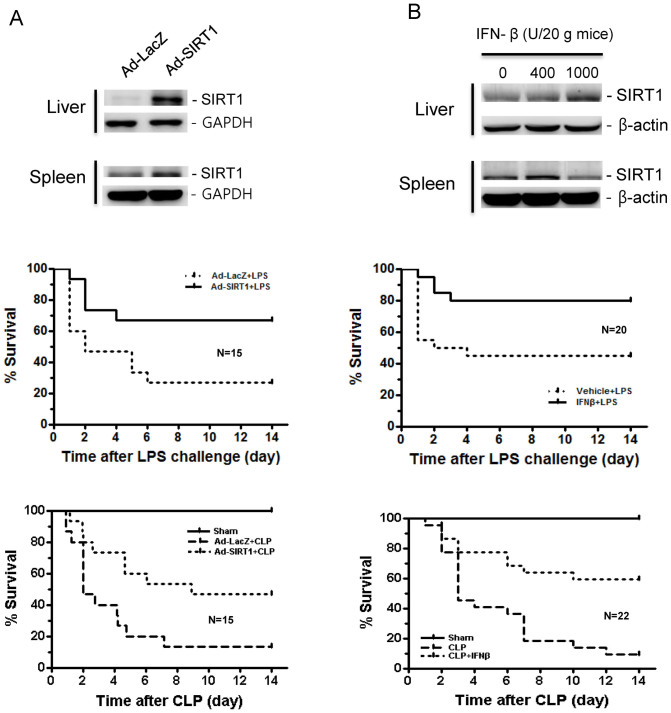
SIRT1-expressing adenovirus infection and IFN-β treatment inhibit LPS-induced lethal endotoxic and septic shock. (A) Survival rate of mice infected with SIRT1-expressing adenovirus in response to LPS challenge or cecal ligation puncture. C57BL/6 mice were randomly grouped and injected with LacZ or SIRT1 expressing adenovirus (1 × 10^10^ MOI). Two days later, the mice were injected with 18 mg/kg body weight of LPS, and ligation and puncture of the cecum (CLP) or sham operation was performed. SIRT1 expression in the liver and spleen of mice infected with SIRT1 expressing adenovirus was checked by western blot (upper panel). The survival rates at different times after LPS challenge (middle panel) or CLP (lower panel) are shown. (B) Survival rate of mice treated with IFN-β in response to LPS challenge or cecal ligation puncture. C57BL/6 mice were injected with vehicle or IFN-β (1000 U/20 g). SIRT1 expression in the liver and spleen of mice treated with IFN-βwas checked by western blot (upper panel). Thirty min later, the mice were injected with 18 mg/kg body weight of LPS, and ligation and puncture of the cecum or sham operation was performed. The survival rates at different times after LPS challenge (middle) or CLP (lower) are shown. Full-length blots are presented in Supplementary Figure S8–S9. The Western blot shown is a representative of three independent experiments.

To test whether SIRT1 and IFN-β protect mice from LPS-induced endotoxemia or cecal ligation and puncture (CLP)-induced sepsis, experiments were performed using mouse models of endotoxemia or polymicrobial sepsis. Mice were intravenously (i.v.) injected with 1 × 10^10^ viral particles of LacZ or SIRT1-expressing adenovirus, and after 48 h the mice were intraperitoneally treated with LPS (18 mg/kg body weight) or subjected to CLP. SIRT1 expressing adenovirus increased SIRT1 expression in the liver and spleen in mice (Fig. 4A, upper). SIRT1 overexpression using SIRT1 expressing adenovirus significantly improved mouse survival rates (66%) after LPS-induced endotoxemia, compared with control LacZ expressing adenovirus-injected mice (26%) as determined on day 6 (Fig. 4A, middle) (log-rank test, *P* < 0.05). SIRT1 overexpression significantly improved mouse survival rates (46%) after CLP-induced sepsis, compared with control LacZ expressing adenovirus-injected mice (13%) as determined on day 6 (Fig. 4A, lower) (log-rank test, *P* < 0.05). IFN-β increased SIRT1 expression in the liver and spleen in mice (Fig. 4B, upper). Pretreatment with IFN-β (1000 units/20 g) 30 min before LPS or CLP challenge significantly improved the survival rates after LPS-induced endotoxemia or CLP-induced sepsis (80% and 63%), compared with control mice(45% and 9%) on day 15 (Fig. 4B) (log-rank test, *P* < 0.05). These results indicate that SIRT1 and IFN-β play important roles in preventing mice from endotoxin-induced lethal shock and polymicrobial sepsis.

### SIRT1 and IFN-β attenuate LPS-induced organ injury

**Figure 5:**
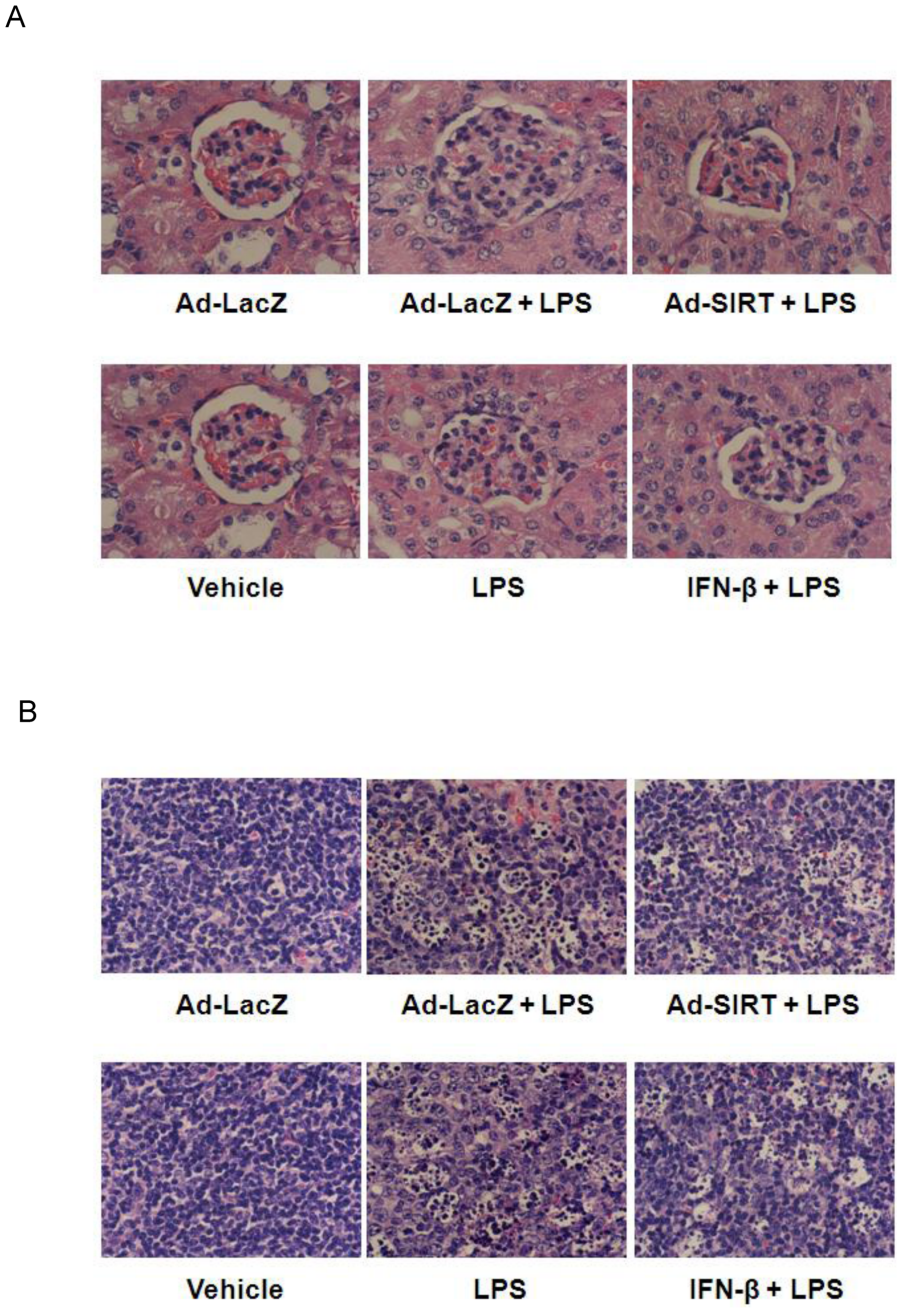
SIRT1-expressing adenovirus infection and IFN-β treatment attenuate organ injury induced by LPS. Morphological changes in mouse kidney (A) and spleen (B) sections infected with LacZ- or SIRT1-expressing adenovirus for 48 h, or treated with vehicle or IFN-β for 30 min before LPS challenge (magnification, ×40). Organ sections were obtained from mice 15 h after treatment with LPS at 18 mg/kg body weight, perfused and fixed with formalin, and stained with hematoxylin and eosin.

Kidneys damaged by LPS challenge were identified by the presence of a reduced Bowman space and induced glomerular hypercellularity (Fig. 5A). LPS-treated mice also showed a morphology of necrotic and apoptotic splenocytes containing small and compact nuclei with multiple nuclear fragments (Fig. 5B). In contrast, the histology of spleen and kidney sections from mice pre-infected with SIRT1-expressing adenovirus or mice treated with IFN-β demonstrated an attenuation of LPS-induced damage to kidney, and necrotic and apoptotic changes in splenocytes (Fig. 5A and B).

### The protective effect of IFN-β on endotoxic shock is dependent on SIRT1 expression

**Figure 6:**
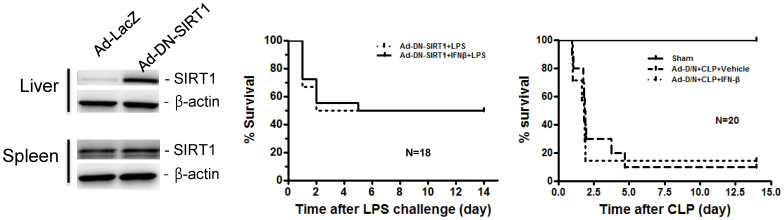
DN SIRT1-expressing adenovirus infection abrogates IFN-β-induced protection against LPS-induced lethal shock. C57BL/6 mice were randomly grouped and injected with DN SIRT1 expressing adenovirus (1 × 10^10^ MOI). Two days later, the mice were injected with vehicle or IFN-β (1000 U/20 g) and 30 min later further injected with 18 mg/kg body weight of LPS. Full-length blots are presented in Supplementary Figure S10. The Western blot shown is a representative of three independent experiments.

To confirm whether the protective effect of IFN-β on LPS-induced endotoxic or CLP-induced septic shock is dependent on SIRT1 expression, we ectopically expressed SIRT1 in mice, using dominant-negative (DN) SIRT1-expressing adenovirus (Fig. 6, left). Mice were i.v. injected with 1 × 10^10^ viral particles of DN SIRT1-expressing adenovirus, and after 48 h the mice were intraperitoneally injected with LPS (18 mg/kg body weight) or subjected to CLP. The infection of mice with DN SIRT1-expressing adenovirus abrogated the protective effect of IFN-β on LPS-induced endotoxic or CLP-induced septic shock (Fig. 6, middle and right). These results indicate that IFN-β protects against endotoxic or septic shock through SIRT1 upregulation.

## Discussion

While it is well established that SIRT1 is a regulator of lifespan extension associated with caloric restriction, energy metabolism, the cell cycle, and differentiation^12^,^13^,^14^,^15^, its role in endotoxic or septic lethality has yet to be fully understood. SIRT1 has been known to inhibit LPS-induced release of proinflammatory cytokines^20^,^21^. In this study, we demonstrated that SIRT1 expression inhibits LPS-induced secretion of proinflammatory cytokines and protects against endotoxemia and sepsis. In addition, we found that IFN-β inhibits LPS-induced secretion of proinflammatory cytokines and protects against endotoxemia and sepsis via SIRT1 upregulation. Thus, our results suggest that SIRT1 or SIRT1 expression-inducing cytokines may be useful targets for pharmacological drug development to protect against sepsis.

There are two signaling mechanisms for TLR4-mediated LPS signal transduction in macrophages, which are divided into MyD88-dependent or MyD88-independent (or TRIF-dependent) signaling pathways^7^,^8^. Inhibition of JAK1 significantly reduced LPS-induced SIRT1 expression, and pretreatment of IFN-β blocking antibody diminished LPS-induced SIRT1 expression. IFN-β alone induced SIRT1 expression in a pattern similar to LPS stimulation. IFN-β-induced SIRT1 expression was also attenuated by treatment with JAK1 inhibitor. Taken together, these data indicate that LPS-induced SIRT1 expression is mediated by the release of IFN-β through the MyD88-independent pathway.

It has been shown that IFN-β knockout mice are resistant to lethal endotoxemia induced by high doses of LPS, and that they have less serum TNF, nitric oxide, and IFN-γ after LPS challenge when compared to wild-type animals^23^. In addition, mice deficient for the IFN-β receptor (IFNAR1) resist LPS-induced lethal endotoxemia^24^. Recent data supports the idea that IFN-β is a potential therapeutic agent that might help to diminish inflammation. IFN-β prevents antigen-induced bronchial inflammation and airway hyperreactivity in mice^25^. IFN-β inhibits the production of TNF-α and IL-1β by 88% and 98%, respectively, whereas the simultaneous production of interleukin-1 receptor antagonist (IL-1Rα) is enhanced two-fold^26^. TNF-α and IL-6 expression are significantly reduced, while IL-10 production is increased after IFN-β treatment^26^. In addition, it has been previously established that IFN-α, another type 1 IFN, has anti-inflammatory action^27^,^28^. As discussed above, the role of IFN-β in inflammation seems to be controversial. The experiments using IFN-β and IFNAR1 knockout models showed that the activation of the IFN-β signaling pathway aggravates inflammation^23^,^24^. However, other reports showed that IFN-β exhibits anti-inflammatory action. Gene knockout can modify cell or organ development and sometimes knockout studies do not reflect the function of knockout genes. Our results demonstrate, in both *in vivo* mouse and *in vitro* macrophage experiments, that IFN-β has anti-inflammatory properties and protects against endotoxic and septic shock.

LPS stimulation of mammalian cells occurs through a series of interactions with several proteins including LBP, CD14, MD-2, and TLR4^29^. TRIF, one of the two TLR4 adaptors, mediates MyD88-independent signaling, activates IRF3, and induces the secretion of IFN-β^7^,^8^. IFN-β transduces its signal via the activation of the JAK-STAT signaling pathway^9^. According to these and our results, IFN-β secreted into extracellular regions binds to its receptor on cell membranes and a signal is transmitted through the JAK-STAT pathway, which induces expression of SIRT1.

Recently, some reports suggested that LPS often induces apoptosis in a cell type-specific manner^30^. But, other reports proposed that LPS is anti-apoptotic in some immune cells^31^. The effect of LPS on apoptosis in macrophages is still controversial^32^. SIRT1 inhibits apoptosis induced by various stimuli^15^,^33^. We also examined the possible relationship between SIRT1 and LPS-induced apoptosis. LPS did not alter expression of p53 and Bcl-2-associated X protein (data not shown). In addition, flow cytometry with annexin V staining also showed no difference in apoptosis between vehicle- and LPS-treated BMDM (data not shown).

This is the first report demonstrating the mechanism of SIRT1 expression in LPS-induced inflammatory reactions and the protective effect of SIRT1 on endotoxemia and sepsis. Our results also suggest that IFN-β and its downstream target, SIRT1, have important roles in anti-inflammatory responses and may be potential targets for treatment of patients with sepsis.

## Methods

### Reagents

IFN-β were purchased from Abcam (Cambridge, MA, USA) and anti-mouse IFN-β was purchased from BioLegend (San Diego, CA, USA). Anti-mouse SIRT1 was purchased from Santa Cruz Biotechnology (Santa Cruz, CA, USA). *Escherichia coli* LPS from serotype 0111:B4, mouse macrophage colony-stimulating factor (MCSF), thioglycollate, anti-β-actin antibody, and anti-α-tubulin antibody were purchased from Sigma-Aldrich (St. Louis, MO, USA). Anti-mouse CD11b-FITC and anti-mouse F4/80-APC were purchased from eBioscience (San Diego, CA, USA). LY294002, wortmannin), SB203580, PD098059, SP600125, PDTC, JAK1 inhibitor, and JAK3 inhibitor were purchased from Calbiochem (San Diego, CA, USA).

### Mice

Male C57BL/6 mice, 20 ~ 24 g in weights, 7 ~ 10 weeks old, were purchased from Damul Science (Daejeon, Korea). The mice were maintained in an environment with controlled temperature (21 ~ 24°C), 12 12 h light-dark cycle, and free access to food and water. We conducted all mouse-related experiments in accordance with the guidelines of the Institutional Animal Care and Use Committee of Chonbuk National University. Our institutional review committee for animal research approved all protocols. For *in vivo* experiments, mice were randomly assigned to control or experimental groups with 10 or more animals for each group.

### Preparation of mouse bone marrow-derived macrophages (BMDMs)

The bone marrow in the femurs and tibias were exposed and flushed with BMDM growth media consisting of RPMI 1640 medium (Welgene, Daejeon, Korea) supplemented with 10% heat-inactivated fetal bovine serum (FBS) (PAA Laboratories GmbH, Pasching, Austria), 100 U/ml penicillin, and 100 μg/mL streptomycin (Invitrogen, Carlsbad, CA, USA). Medium containing bone marrow cells was centrifuged for 5 min at 400 × *g*. The bone marrow cell were cultured in the above medium containing MCSF (10 ng/mL), and allowed to differentiate for 7 days. The differentiation into macrophages were confirmed by flow cytometry by blocking the cells with anti-Fcγreceptor antibody (eBioscience, San Diego, CA, USA) and staining the cells with saturating amounts of the FITC-conjugated anti-mouse CD11b and APC-conjugated anti-mouse F4/80 antibodies (eBioscience). Over 95% of cells were double positive for F4/80 and CD11b macrophage surface markers (Fig. 1A).

### Preparation of the recombinant adenovirus

SIRT1 or dominant negative (DN)-SIRT1 expressing adenovirus was prepared according to the method described previously^33^.

### LPS-induced endotoxemia model

Age-matched mice (7 ~ 9 weeks of age) were intravenously injected with 1 × 10^10^ particles of recombinant adenoviral construct expressing SIRT1, DN-SIRT1 or LacZ on day −2. On day 0, the mice underwent an intraperitoneal injection of 18 mg LPS/kg of body weight.

### Cecal ligation and puncture

Cecal ligation and puncture was performed according to the methods described previously with some modifications^34^. Mice were anesthetized using isoflurane (induction 3%, maintenance 1.5%, oxygen flow 3 L/min) and subjected to a 1 cm ventral midline abdominal incision. The cecum was then exposed, ligated with 4-0 silk suture just distal to the ileocecal valve (comprising 70% of the cecum and sparing the cecal vessels) to avoid intestinal obstruction, and punctured-through with a 24-gauge needle. The punctured cecum was gently squeezed to expel a 1–2-mm droplet of fecal material and returned to the abdominal cavity. The incision was then closed in layers using 4-0 surgical sutures. Mice were fluid resuscitated with prewarmed normal saline (500 μL) intraperitoneally, immediately after the procedure. Sham animals underwent the same procedure except for ligation and puncture of the cecum. All experiments were carried out at the same time of the day.

### Western blotting

BMDMs were rinsed twice with ice-cold PBS then lysed in mammalian cell lysis solution (Thermo Scientific, Waltham, MA, USA) containing protease inhibitor cocktail (Roche, Indianapolis, IN, USA) for 30 min, and centrifuged to separate the soluble supernatants. Tissues collected from mice were immediately stored at −80°C. Frozen tissues were homogenized, lysed in tissue lysis solution (Thermo Scientific), and centrifuged at 10,000 × *g* at 4°C for 10 min. Protein extracts (20 μg) were separated by 10% sodium dodecyl sulfate-polyacrylamide gel electrophoresis at 100 V with a running time of 120 min, and transferred to polyvinylidene fluoride membranes. Membranes were blocked with 3% nonfat milk in Tris-buffered saline (pH 7.4) with 0.1% Tween 20 for 1 h at room temperature, and probed overnight at 4°C with primary antibodies against SIRT1, β-actin, and α-tubulin, respectively. After washing, the membranes were further incubated with species-specific HRP-conjugated secondary antibody, for 50 min at room temperature. Immunoreactive proteins were detected by using an enhanced chemiluminescence detection system (G&E Amersham Life Science, Arlington Heights, IL, USA) according to the manufacturer's recommendations.

### RNA extraction and quantitative real time PCR

Total RNA was extracted using RNeasy mini kit (QIAGEN, Germantown, MD, USA) according to manufacturer's instructions. Real-time PCR primer sets for mouse-SIRT1, and mouse-18 small subunit ribosomal RNA (18S) were purchased from SABiosciences (Frederick, MD, USA). The 18S was used as an internal control. Total RNA was treated with DNase I to remove residual DNA. RNA was treated with RNase-free DNase for 20 min at room temperature before reverse transcription. RNA concentrations were determined by OD_260_ absorbance. Reverse transcription was performed with 1 μg of RNA in a 20 μL volume by using the ImProm-II™ Reverse Transcription System (Promega). Real-time quantitative PCR was then performed in a 20 μL volume using SYBR Green reaction mix (Stratagene, San Diego, CA, USA) with 10 μM primer set and 1 μL cDNA in a 7900HT Real-time PCR machine (Applied Biosystems, Foster City, CA, USA). PCR conditions consisted of 2 min at 50°C and 10 min hot start at 95°C, followed by 40 cycles of 15 s at 95°C and 1 min at 60°C. The average threshold cycle (Ct) for each gene was determined from triplicate reactions, and the levels of gene expression relative to 18S were determined.

### Cytokine measurements

TNF-α, IL-6, monocyte chemoattractact protein (MCP)-1, and IL-10 in BMDM culture medium were measured by using specific enzyme-linked immunosorbent assay (ELISA) kits (Koma Biotechnology, Seoul, South Korea), according to the manufacturer's instructions.

### Histological examination

Tissues of kidney and spleen were fixed with 10% formaldehyde and were embedded in paraffin. Paraffin sections 4-μm thick were stained with hematoxylin and eosin for examination by light microscopy.

### Statistical analysis

Data were presented as mean ± standard error. Student's *t* test was used to determine the significance of values and *P* < 0.05 were considered significant. All survival comparisons were made by the log-rank test and *P* values were derived from one-tailed tests.

## Author Contributions

The study was performed in collaboration between all authors. S.-I.L. and M.-K.H. designed methods and experiments, analyzed the data, interpreted the results and wrote the paper. E.-K.S. carried out the laboratory experiments about *in vitro* analysis and directed the exprements carried out by C.-H.Y. and J.-H.Y. C.-H.Y. and J.-H.Y. co-designed experiments, performed repeated experiments, and discussed analyses. J.-J.H. performed some western blot experiments. All authors have contributed, seen and approved the manuscript.

## Supplementary Material

Supplementary Information Suppoting Material
