# Supplementary material for: Interferon β protects against lethal endotoxic and septic shock through SIRT1 upregulation
Source: Sci Rep. 2014 Feb 27;4:4220. doi: 10.1038/srep04220 (PMC3936230; doi:10.1038/srep04220)
Supplement: Supplementary Information — Suppoting Material [file srep04220-s1.pdf]

SUPPORTING MATERIAL

Interferon β protects against lethal endotoxic and septic shock through SIRT1 upregulation

Chae-Hwa Yoo[*, †], Ji-Hyun Yeom[*, †], Jin-Ju Heo[*], Eun-Kyung Song[*,¶], Sang-Il Lee[‡, ¶], and Myung-Kwan Han[*, §, ¶]

[*]Department of Microbiology, [§]Institute for Medical Science, Chonbuk National University Medical School, Jeonju 561-182, Republic of Korea

[‡]Department of Internal Medicine, Institute of Health Science, Gyeongsang National University College of Medicine, Jinju 660-702, Republic of Korea

[†]Both authors contributed equally to this work

[¶]Correspondence: E.-K. Song, S.-I. Lee or M.-K. Han, Department of Microbiology, Chonbuk National University Medical School, Jeonju 561-182, Republic of Korea. E-mail: silverysk@hanmail.net (E.-K.S.), goldgu@gnu.ac.kr (S.-I.L.) or iamtom@chonbuk.ac.kr (M.-K.H.)

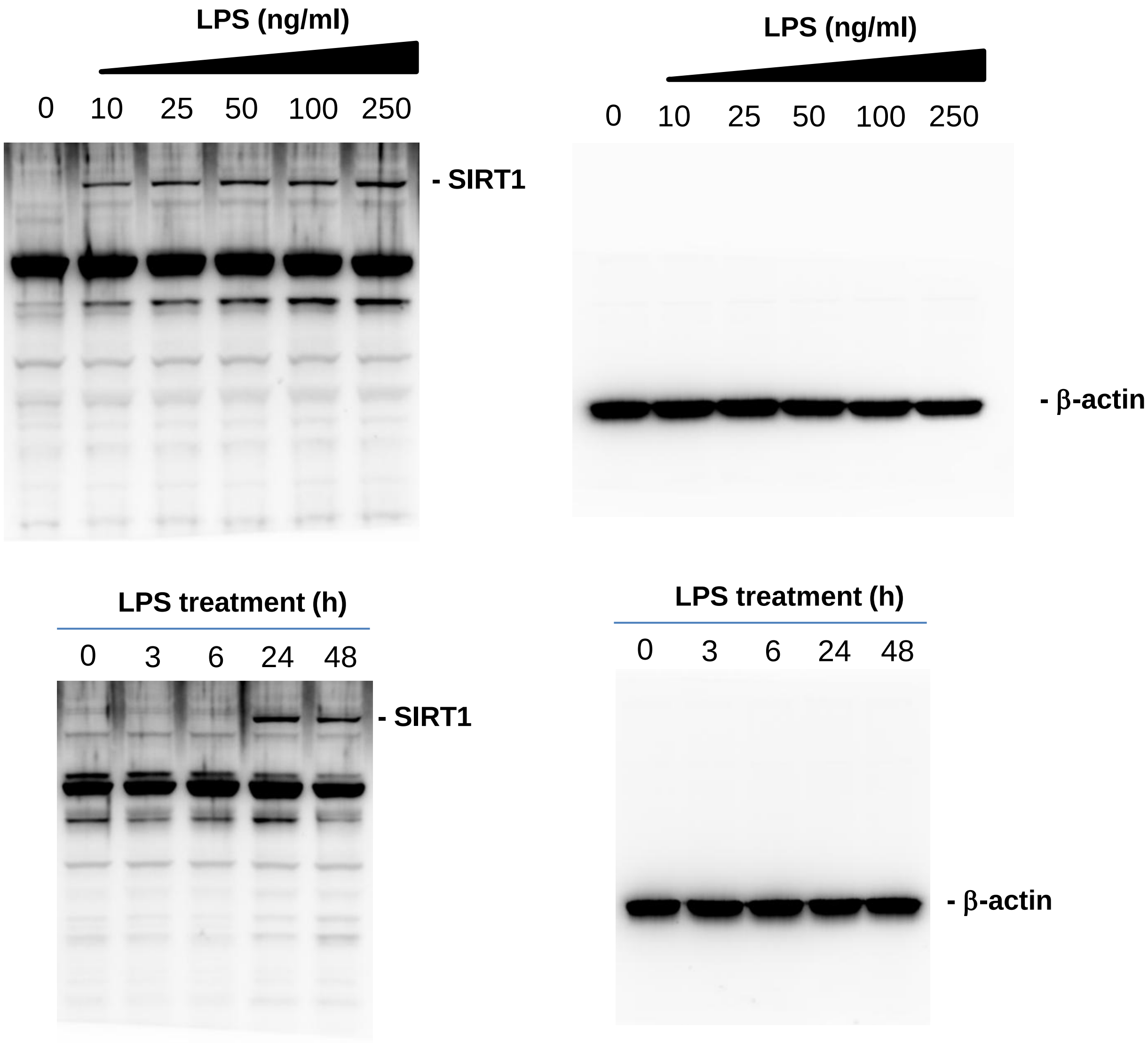

Supplementary Figure S1 Full-length blots for Figure 1B

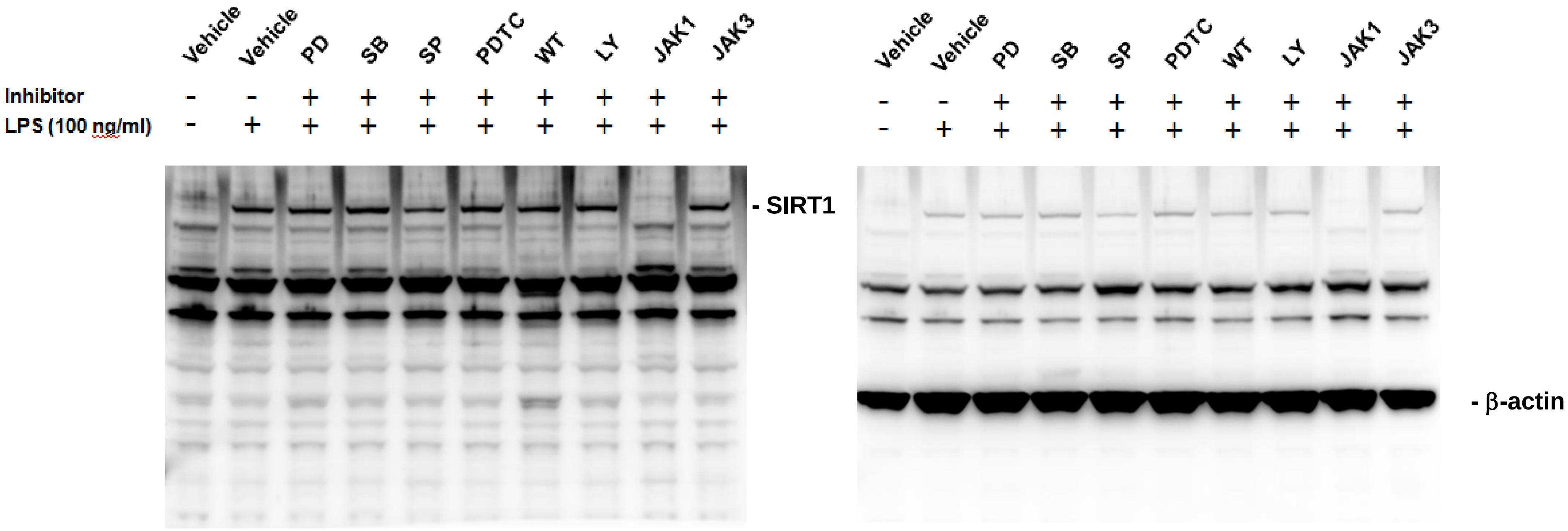

Supplementary Figure S2 Full-length blots for Figure 1D

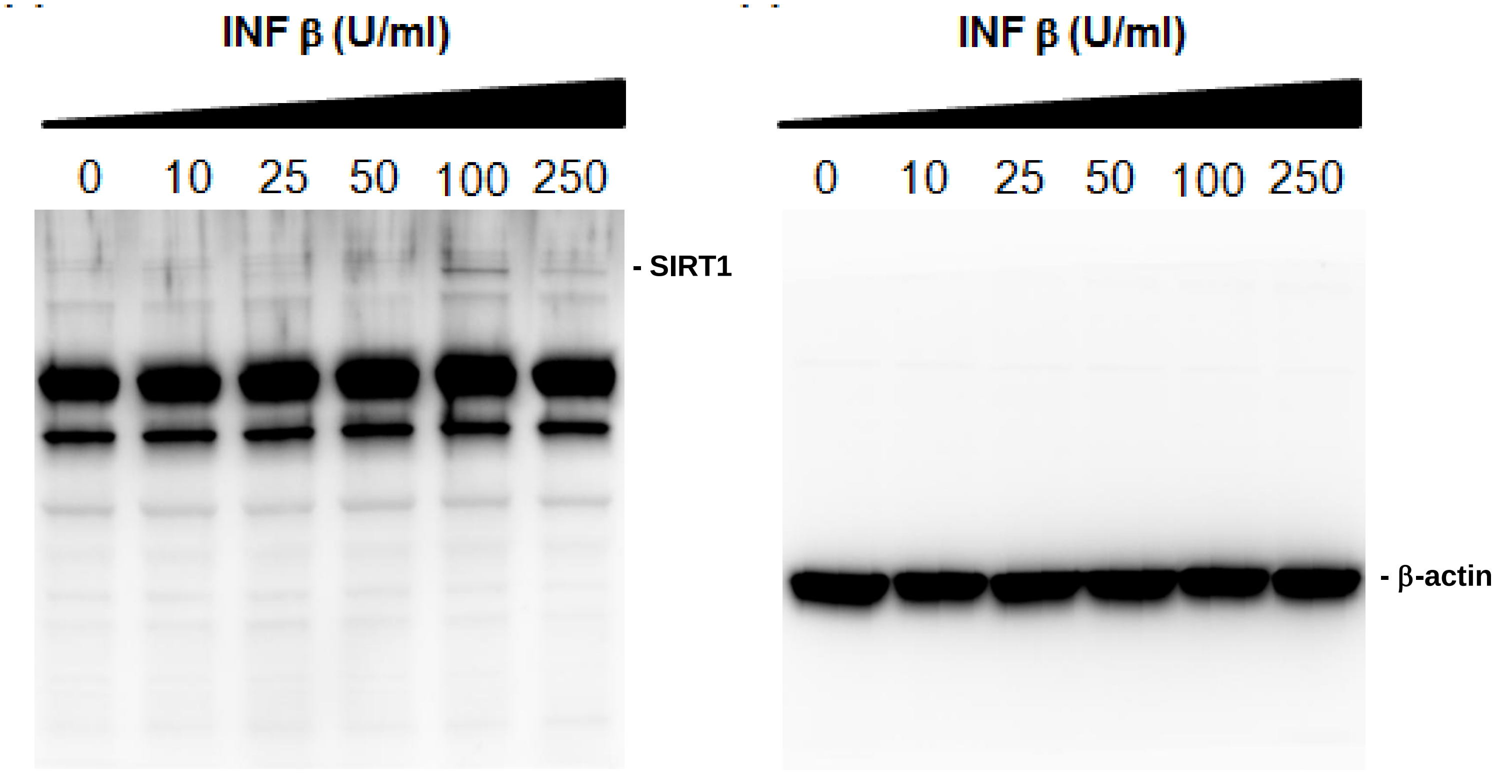

Supplementary Figure S3 Full-length blots for Figure 2A

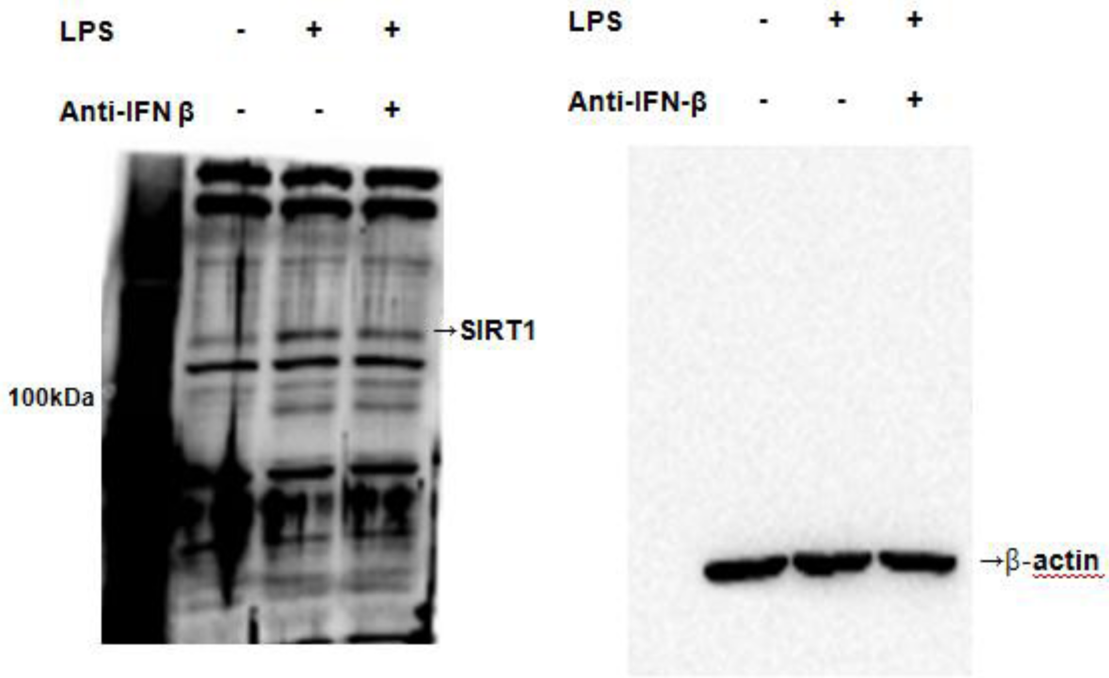

Supplementary Figure S4 Full-length blots for Figure 2B

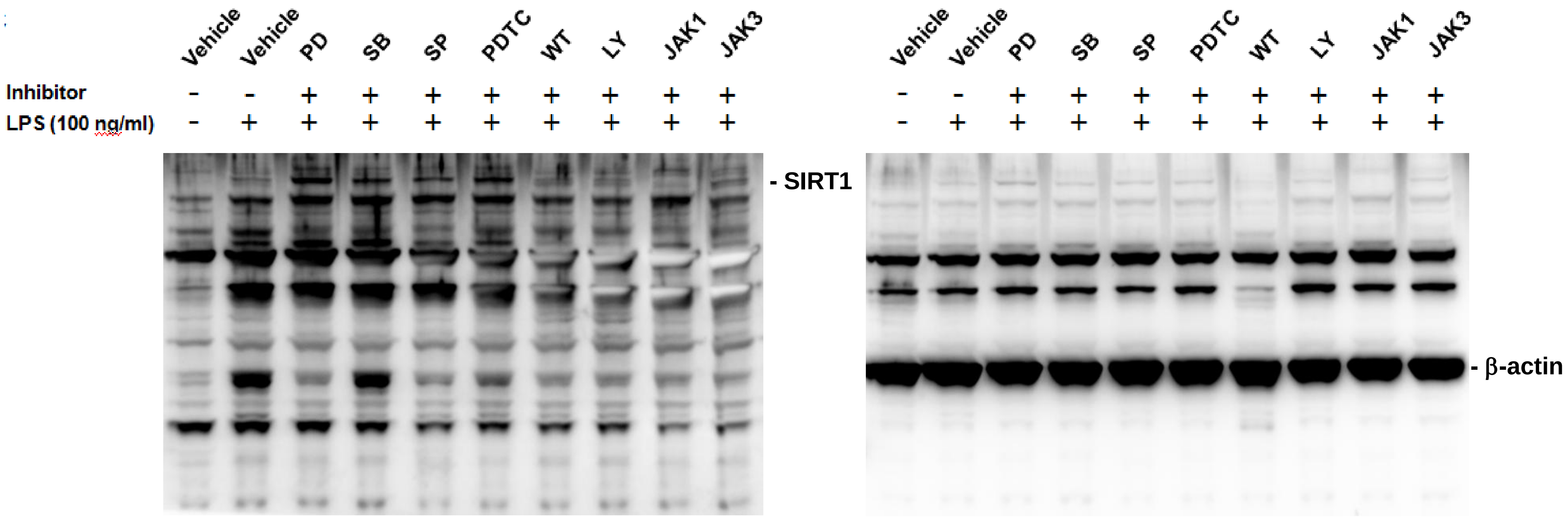

Supplementary Figure S5 Full-length blots for Figure 2C

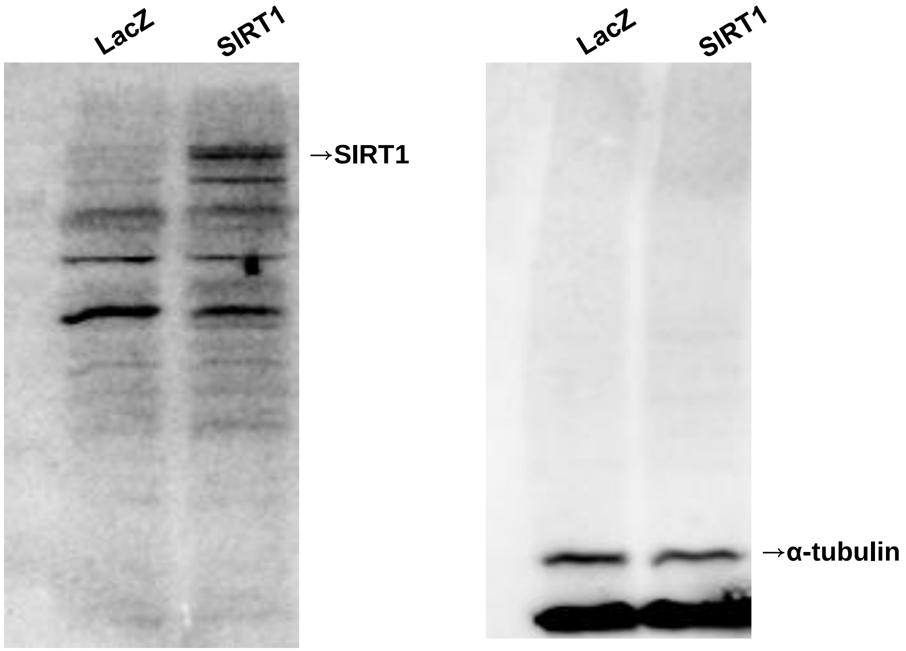

Supplementary Figure S6 Full-length blots for Figure 3A

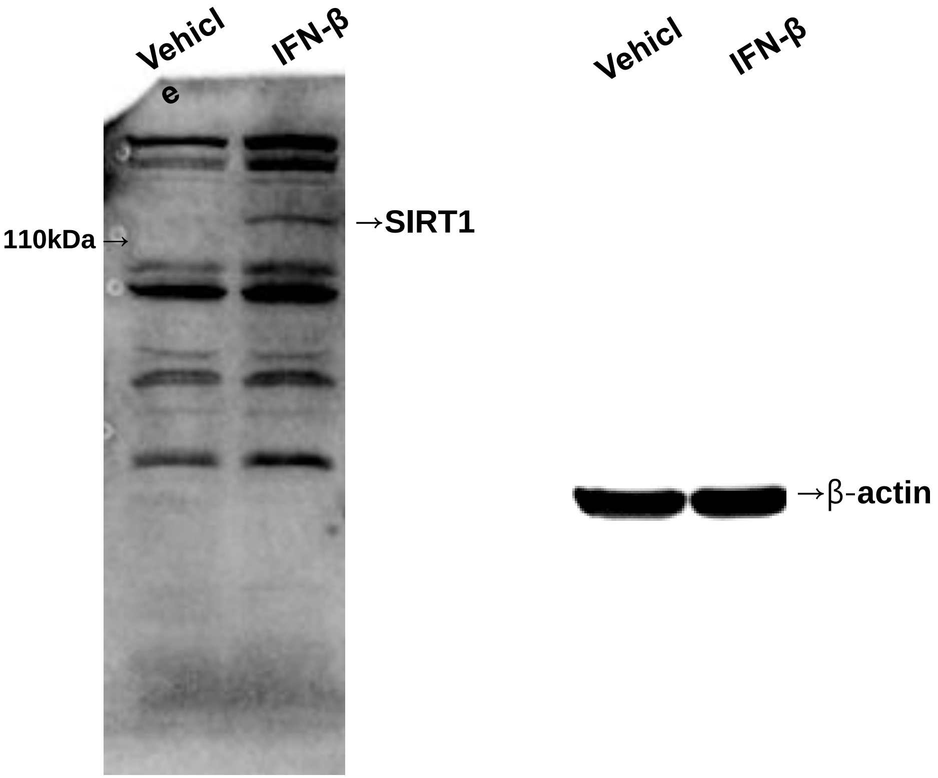

Supplementary Figure S7 Full-length blots for Figure 3B

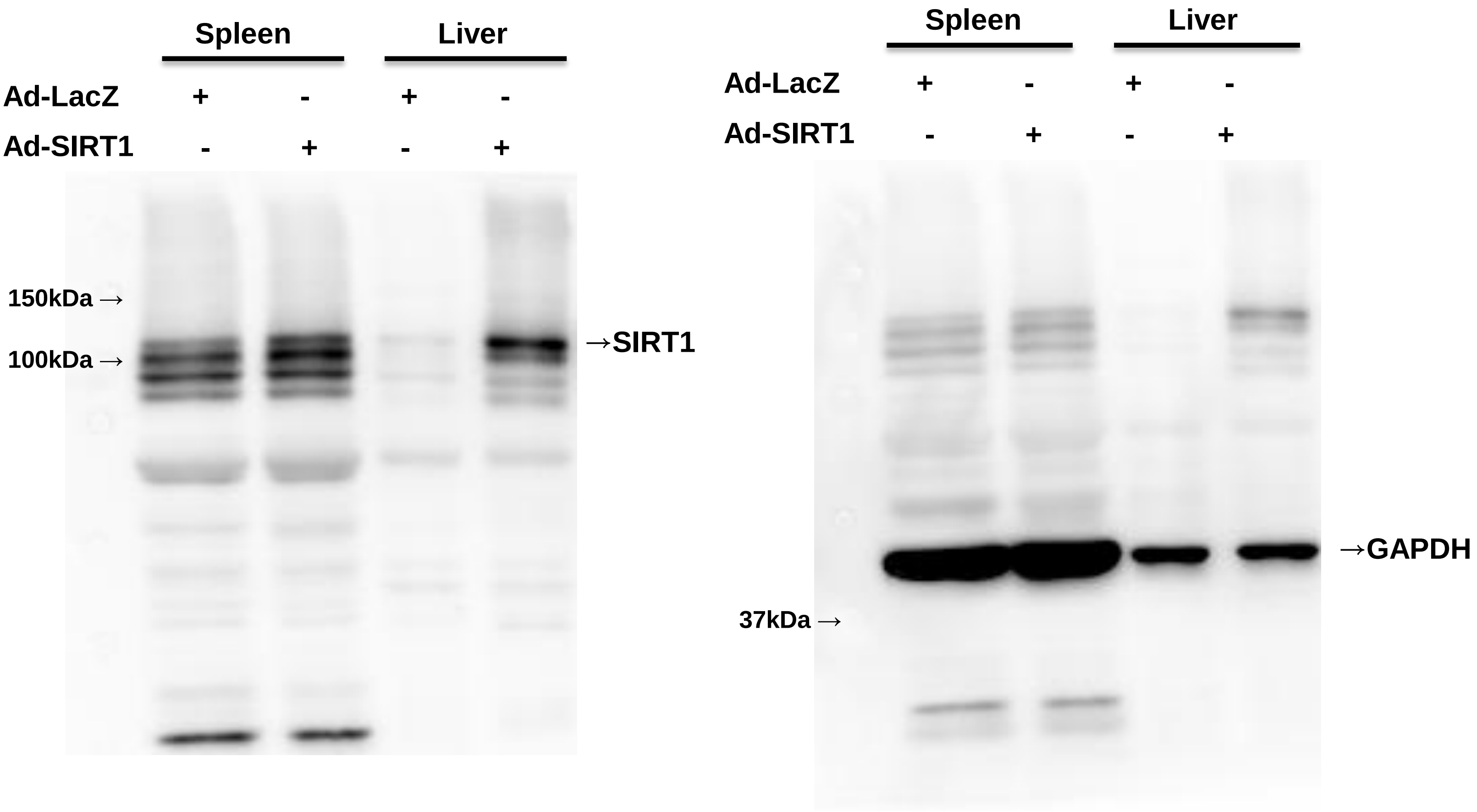

Supplementary Figure S8 Full-length blots for Figure 4A

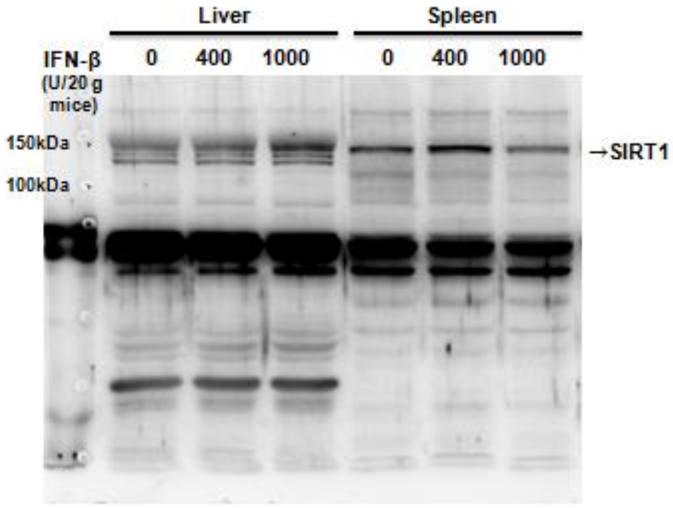

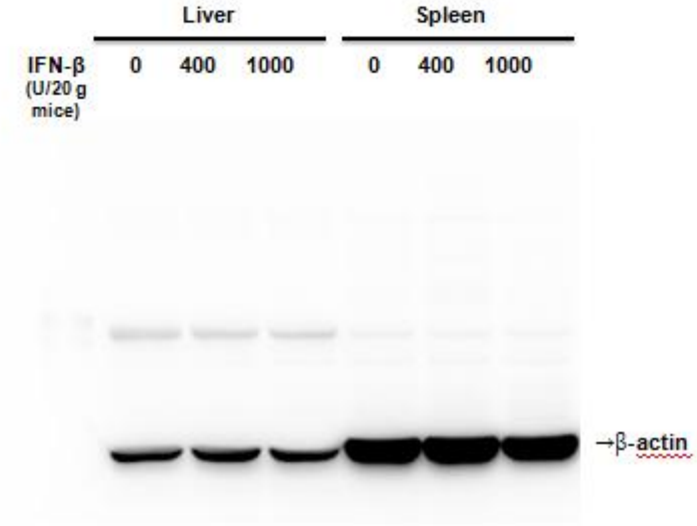

Supplementary Figure S9 Full-length blots for Figure 4B

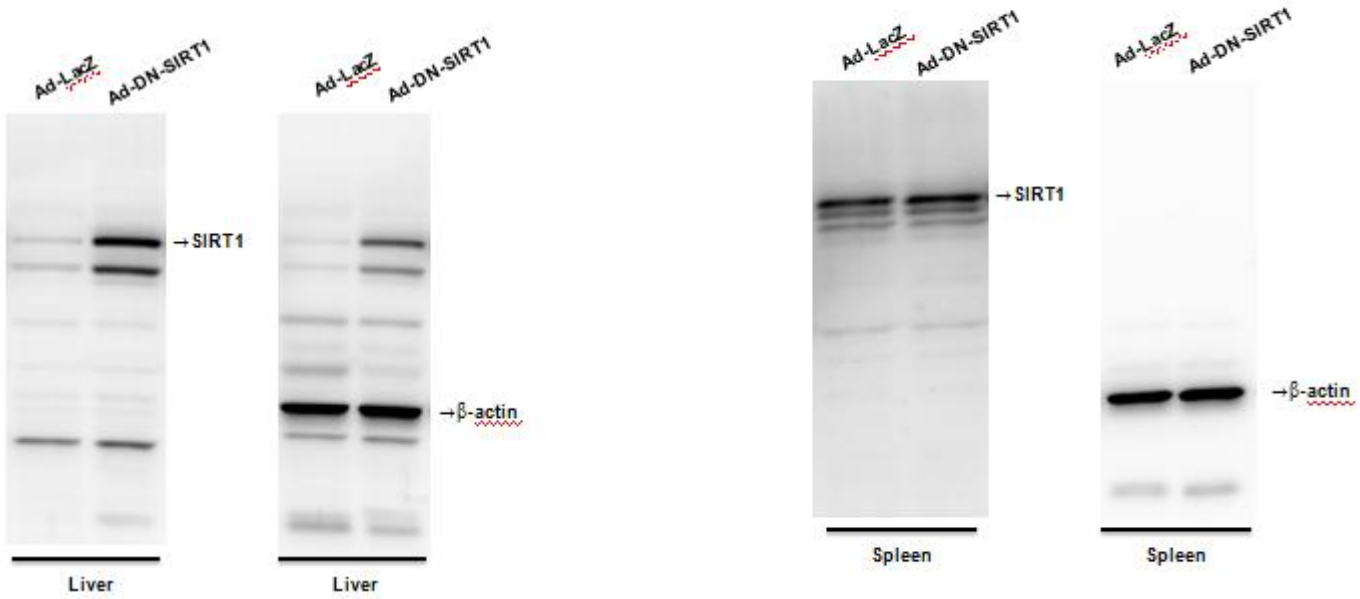

Supplementary Figure S10 Full-length blots for Figure 6
